# Effect of sublethal dose of chloramphenicol on biofilm formation and virulence in *Vibrio parahaemolyticus*

**DOI:** 10.3389/fmicb.2023.1275441

**Published:** 2023-09-26

**Authors:** Miaomiao Zhang, Liyan Cai, Xi Luo, Xue Li, Tingting Zhang, Fei Wu, Yiquan Zhang, Renfei Lu

**Affiliations:** ^1^Department of Clinical Laboratory, Nantong Third People's Hospital, Affiliated Nantong Hospital 3 of Nantong University, Nantong, China; ^2^Physical Examination Center, Nantong Third People's Hospital, Affiliated Nantong Hospital 3 of Nantong University, Nantong, China; ^3^School of Medicine, Nantong University, Nantong, China

**Keywords:** *Vibrio parahaemolyticus*, chloramphenicol, gene expression, biofilm, virulence

## Abstract

*Vibrio parahaemolyticus* isolates are generally very sensitive to chloramphenicol. However, it is usually necessary to transfer a plasmid carrying a chloramphenicol resistance gene into *V. parahaemolyticus* to investigate the function of a specific gene, and the effects of chloramphenicol on bacterial physiology have not been investigated. In this work, the effects of sublethal dose of chloramphenicol on *V. parahaemolyticus* were investigated by combined utilization of various phenotypic assays and RNA sequencing (RNA-seq). The results showed that the growth rate, biofilm formation capcity, c-di-GMP synthesis, motility, cytoxicity and adherence activity of *V. parahaemolyticus* were remarkably downregulated by the sublethal dose of chloramphenicol. The RNA-seq data revealed that the expression levels of 650 genes were significantly differentially expressed in the response to chloramphenicol stress, including antibiotic resistance genes, major virulence genes, biofilm-associated genes and putative regulatory genes. Majority of genes involved in the synthesis of polar flagellum, exopolysaccharide (EPS), mannose-sensitive haemagglutinin type IV pilus (MSHA), type III secretion systems (T3SS1 and T3SS2) and type VI secretion system 2 (T6SS2) were downregulated by the sublethal dose of chloramphenicol. Five putative c-di-GMP metabolism genes were significantly differentially expressed, which may be the reason for the decrease in intracellular c-di-GMP levels in the response of chloramphenicol stress. In addition, 23 genes encoding putative regulators were also significantly differentially expressed, suggesting that these regulators may be involved in the resistance of *V. parahaemolyticus* to chloramphenicol stress. This work helps us to understand how chloramphenicol effect on the physiology of *V. parahaemolyticus*.

## Introduction

*Vibrio parahaemolyticus* (*V. parahaemolyticus*), the leading cause of seafood-related gastroenteritis, produces multiple virulence factors playing different roles in its pathogenesis, including thermostable direct hemolysin (TDH), TDH-related hemolysin (TRH), type III secretion systems (T3SS1 and T3SS2), type VI secretion systems (T6SS1 and T6SS2), lipopolysaccharide and extracellular proteases (Cai and Zhang, 2018; Osei-Adjei et al., 2018; Li et al., 2019; Pazhani et al., 2021). Both TDH and TRH have hemolytic activity, but only TDH can cause β-type hemolysis on Wagatsuma agar called the Kanagawa phenomenon (Xu et al., 1994; Cai and Zhang, 2018). TDH also possesses the lethal toxicity, cytotoxicity, and enterotoxicity (Park et al., 2004; Hiyoshi et al., 2010; Raghunath, 2014; Cai and Zhang, 2018). T3SS, a needle-like injection system, can manipulate host cell functioning by injecting toxic proteins into host cells (Pais et al., 2023). Both T3SS1 and T3SS2 are required for the full virulence of *V. parahaemolyticus* (Hiyoshi et al., 2010; Matsuda et al., 2012; Zhou et al., 2013; Gu et al., 2020; Hu et al., 2021; Lin et al., 2021). However, T3SS1 plays significant roles in cytotoxic activity against various cell lines and lethal activity in mice, whereas T3SS2 is the major contributor to enterotoxicity of *V. parahaemolyticus* (Hiyoshi et al., 2010). Moreover, T6SS1 possesses antibacterial activity and thus is required for the environmental fitness of *V. parahaemolyticus*, whereas T6SS2 plays a role in cell adhesion (Yu et al., 2012; Salomon et al., 2013; Coulthurst, 2019).

*Vibrio parahaemolyticus* is often found in the form of biofilms, which are matrix-enclosed, surface associated communities (Yildiz and Visick, 2009; Ruhal and Kataria, 2021). Some specific structures such as flagella, exopolysaccharide (EPS) and type IV pili are required for the mature biofilm formation of *V. parahaemolyticus* (Enos-Berlage et al., 2005; Shime-Hattori et al., 2006; Yildiz and Visick, 2009; Jung et al., 2019; Li et al., 2020; Ruhal and Kataria, 2021; Liu et al., 2022). *V. parahaemolyticus* expresses two kinds of flagellar systems, a polar flagellum for swimming in liquid and lateral flagella for swarming on the surface (McCarter, 2004). Loss of polar flagellum prevents *V. parahaemolyticus* to form mature biofilms (Enos-Berlage et al., 2005). The *cpsA-K* and *scvA-O* gene clusters are responsible for the synthesis of EPS in *V. parahaemolyticus* (Liu et al., 2022). Strains without *cps* genes form smooth colonies on the agar plate, whereas those without *scv* genes form wrinkly colonies (Liu et al., 2022). The wrinkly phenotype spreader has stronger biofilm formation capacities than the smooth phenotype strain (Wu et al., 2022). *V. parahaemolyticus* possesses two kinds of type IV pili, mannose-sensitive haemagglutinin type IV pili (MSHA) and chitin-regulated pili (ChiRP) (Shime-Hattori et al., 2006). MSHA is required for bacterial attachment, whereas ChiRP plays a role in bacterial agglutination (Shime-Hattori et al., 2006). *V. parahaemolyticus* biofilm formation is also strongly correlated with the contents of proteins and DNA in the extracellular polymeric substance (Li et al., 2020). In addition, some regulators such as QsvR (Zhang et al., 2019, 2023b), H-NS (Sun et al., 2014; Zhang et al., 2018; Xue et al., 2022) and ToxR (Chen et al., 2018; Zhang et al., 2018) and some regulatory processes such as quorum sensing (QS) pathways (Wang et al., 2013; Zhang et al., 2019; Liu et al., 2021; Zhang et al., 2023b) and cyclic di-GMP (c-di-GMP) (Kim and McCarter, 2007; Tamayo et al., 2007; Kimbrough et al., 2020; Kimbrough and McCarter, 2021; Zhong et al., 2022) are involved in the regulation of virulence and biofilm formation of *V. parahaemolyticus*.

Almost all *V. parahaemolyticus* isolates exhibited multiple antibiotics resistances, especially to ampicillin and colistin (Amalina et al., 2019; Mok et al., 2021; Sun et al., 2022; Zaafrane et al., 2022). Previously, two class 1 integrons, *dfrA14*-*bla*_VEB-1_-*aadB* and *bla*_VEB-1_-*aadB*-*arr2*-*cmlA*-*bla*_OXA-10_-*aadA1*, which are strongly associated with the resistance to multiple antibiotics such as ampicillin, ceftazidime and cefotaxime, were detected in *V. parahaemolyticus* (Lei et al., 2020). Additionally, a total of 12 resistance nodulation cell division (RND)-type efflux transporters playing important roles in intrinsic resistance to antibiotics were estimated in *V. parahaemolyticus*, among which VmeAB, VmeCD, VmeEF, VmeYZ have already been characterized (Matsuo et al., 2007, 2013). Majority of *V. parahaemolyticus* isolates are sensitivity to chloramphenicol (Amalina et al., 2019; Lei et al., 2020; Mok et al., 2021; Zaafrane et al., 2022). However, a novel chloramphenicol acetyltransferase gene, *catC*, is distributed among the *V. parahaemolyticus* strains, and heterologously expressing this gene was able to enhance chloramphenicol resistance of *E. coli* (Zhang et al., 2019).

The suicide plasmid pDS132 and the L-arabinose induced plasmid pBAD33 are widely used to construct specific mutants and complemented mutants, respectively (Guzman et al., 1995; Philippe et al., 2004). Both pDS132 and pBAD33 harbor a chloramphenicol resistance gene, but *V. parahaemolyticus* strains transformed with pDS132 or pBAD33 were still unable to grow in the conditions containing chloramphenicol higher than 5 μg/mL (Sun et al., 2014; Zhang et al., 2018, 2023b; Zhang Y. et al., 2023). Therefore, chloramphenicol probably has great effect on the growth and physiology of *V. parahaemolyticus*. In this work, we aimed to investigate the sublethal dose of chloramphenicol on the physiology of *V. parahaemolyticus*. Notably reductions in growth, motility, biofilm formation, c-di-GMP production, cytoxicity and adhesion activity were evident in the response to sublethal doses of chloramphenicol. Sublethal dose of chloramphenicol greatly affected the expression of 650 genes in *V. parahaemolyticus*, including the antibiotic resistance genes, major virulence genes, biofilm-associated genes and putative regulatory genes. This work helps us to understand how chloramphenicol effect on the physiology of *V. parahaemolyticus*.

## Materials and methods

### Growth conditions

*Vibrio parahaemolyticus* RIMD2210633, which was isolated from a diarrhea patient in 1996 (Makino et al., 2003) and kindly provided by Dr. Dongsheng Zhou from Beijing Institute of Microbiology and Epidemiology, was used throughout in this work. Approximately 20 μL glycerol stock of bacterial cells was inoculated into 5 mL 2.5% (w/v) Bacto heart infusion broth (HI broth; BD Bioscience, USA), followed by incubated at 37°C with shaking at 200 rpm for 12 h. The resultant cultures were diluted 50-fold into 5 mL of fresh HI broth for the second round of growth, which were grown to an OD_600_ value of 1.5. The resultant cultures were then diluted 1000-fold into 5 mL of fresh HI broth for a third round of growth, and were harvested at an OD_600_ value of approximately 1.5. When necessary, the medium was supplemented with different concentrations of chloramphenicol (0, 0.2, 0.3 or 0.4 μg/mL).

### Growth inhibition assay

The second round of bacterial cell cultures was diluted 1000-fold into 10 mL HI broth containing 0, 0.2, 0.3 or 0.4 μg/mL chloramphenicol in a glass tube, and allowed to grow continuously at 37°C with shaking at 200 rpm. Three biological replicates were set for each concentration. The growth curves were created by monitoring the OD_600_ values of each culture at 1 h intervals.

### Biofilm formation assay

Biofilm formation capacities of *V. parahaemolyticus* under different concentrations of chloramphenicol were assessed by the crystal violet (CV) staining method, which was performed similarly as previously described (Fang et al., 2013; Zhang et al., 2021). Briefly, the second round of bacterial cell cultures was diluted 50-fold into 2 mL Difco marine (M) broth 2,216 (M broth; BD Bioscience, USA) containing 0, 0.2 or 0.4 μg/mL chloramphenicol in a 24-well cell culture plate, and allowed to grow at 30°C with shaking at 150 rpm for 48 h. The surface-attached cells were washed gently three times with deionized water after removing the planktonic cells, incubated at 80°C for 15 min to fix the biofilm cells, and then stained with 3 mL of 0.1% CV for 30 min. The CV stained biofilm cells were washed for another three times with deionized water. The bound CV was dissolved with 20% acetic acid, and the OD_570_ values were measured as an index of CV staining.

### Colony morphology

Colony morphology assay was performed similarly as previously described (Zhang et al., 2021). Briefly, the second round of bacterial cell cultures was diluted 50-fold into 2 mL M broth in a 24-well cell culture plate, and allowed to grow without shaking at 30°C for 48 h. The cell culture was mixed thoroughly, and then 2 μL of the culture was spotted onto a HI agar containing 0, 0.2 or 0.4 μg/mL chloramphenicol, followed by incubated at 30°C for 48 h.

### Opaque (OP)-translucent (TR) colony phenotypes

The OP-TR colony phenotypes were detected as previously described (Zhang et al., 2023b). Briefly, a small amount of overnight cell culture in HI broth was streaked onto a HI plate containing 0, 0.2 or 0.4 μg/mL chloramphenicol, and then statically incubated at 37°C for 24 h.

### Swimming motility

Swimming motility assay was performed as previously described (Wu et al., 2022). Briefly, 2 μL of the second round of bacterial cell culture were inoculated into a swimming plate [1% Oxoid tryptone, 2% NaCl (Merck, Germany), and 0.5% Difco Noble agar (BD Biosciences, USA)] containing 0, 0.2 or 0.4 μg/mL chloramphenicol. The swimming plate was statically incubated at 37°C. Starting from the 3rd hour after incubation, the diameter of swimming area was measured per hour until the 7th hour.

### Swarming motility

Swarming motility assay was performed as previously described (Wu et al., 2022). Briefly, 2 μL of the second round of bacterial culture were spotted on a solid swarm plate [2.5% Bacto heart infusion, 1.5% NaCl (Merck), and 2.0% Difco noble agar (BD Bioscience)] containing 0, 0.2 or 0.4 μg/mL chloramphenicol. The diameters of swarming zones were measured at the 24th, 48th, and 72th hours after incubation statically at 37°C.

### Intracellular c-di-GMP quantification

Intracellular c-di-GMP levels were measured as previously described (Wu et al., 2022). Briefly, the second round of bacterial culture was diluted 1000-fold into 5 mL of HI broth containing 0, 0.2 and 0.4 μg/mL chloramphenicol, respectively, and incubated at 37°C with shaking to an OD_600_ value of 1.5. Bacterial cells were harvested and resuspended in 2 mL ice-cold phosphate buffered saline (PBS), and then incubated at 100°C for 5 min, followed by sonicated for 15 min (power 100%, frequency 37 kHz) in an ice-water bath. The supernatant containing c-di-GMP was collected, and the pellet was resuspended in 2 mL ice-cold PBS and re-extracted for another two times. The intracellular c-di-GMP levels were determined with a c-di-GMP Enzyme-linked Immunosorbent Assay (ELISA) Kit (Mskbio, Beijing, China). In addition, total protein in the supernatant was also determined by a Pierce BCA Protein Assay kit (ThermoFisher Scientific, USA). Intracellular c-di-GMP levels were expressed as pmol/g protein.

### Cytotoxicity assay

Cytotoxicity assay was performed as previously described (Sun et al., 2014). Briefly, the second round of bacterial culture was diluted 1000-fold into 5 mL of HI broth containing 0, 0.2 and 0.4 μg/mL chloramphenicol, respectively, and incubated at 37°C with shaking to an OD_600_ value of 1.5. Bacterial cells were harvested, washed and then serially diluted with the pre-warmed Dulbecco’s modified Eagle’s medium (DMEM) lacking phenol red for colony forming unit (CFU) measurement and infection. HeLa cells were infected with 10^6^ CFU of bacteria for 3 h at a multiplicity of infection (MOI) of 2.5. The release of lactate dehydrogenase (LDH) was quantified with a CytoTox 96^®^ Non-Radioactive Cytotoxicity Assay kit (Promega, USA) according to the manufacturer’s instructions.

### Adhesion assay

Adhesion assay was performed similarly as previously described (Zhang et al., 2017). Briefly, HeLa cell monolayers were maintained in DMEM supplemented with 10% fetal bovine serum (FBS, Invitrogen) at 37°C with 5% CO_2_. The second round of bacterial culture was diluted 1,000-fold into 5 mL of HI broth containing 0, 0.2 and 0.4 μg/mL chloramphenicol, respectively, and incubated at 37°C with shaking to an OD_600_ value of 1.5. Bacterial cells were collected, washed, and then re-suspended in DMEM. The cell monolayers were infected at multiplicity of infection (MOI) of 1:10. Bacterial cells were also added to empty wells to determine the amount of input as the final total number of *V. parahaemolyticus*. After incubation for 90 min, the monolayers were washed thrice with PBS and lysed with 1% Triton X-100. The lysates and input bacteria were serially diluted 10-fold and counted on LB agar plates. Percent adherence was calculated as bacterial cells adhered/input bacterial cells.

### RNA sequencing (RNA-seq)

The second round of bacterial culture was diluted 1000-fold into 5 mL of HI broth containing 0 and 0.2 μg/mL chloramphenicol, respectively, and incubated at 37°C with shaking to an OD_600_ value of 1.5. Bacterial cells were harvested, and total RNA was extracted using TRIzol Reagent (Invitrogen, USA). RNA concentrations were measured using a Nanodrop 2000, and RNA integrity was evaluated using the agarose gel electrophoresis (Wu et al., 2022). rRNA removal and mRNA enrichment were performed using an Illumina/Ribo-Zero™ rRNA Removal Kit (bacteria) (Illumina, USA). cDNA library construction and sequencing were performed on an Illumina Hiseq platform at the GENEWIZ Biotechnology Co. Ltd. (Suzhou, China). The significantly differentially expressed genes (DEGs) were analyzed using the DESeq2 (V1.6.3) software with at least 2-fold changes in the ratio of mRNA levels (test/reference) and a *p* value of <0.05.

### Quantitative PCR (qPCR)

qPCR assays were performed similarly as previously described (Gao et al., 2011). Briefly, the second round of bacterial culture was diluted 1000-fold into 5 mL of HI broth containing 0 and 0.2 μg/mL chloramphenicol, respectively, and incubated at 37°C with shaking to an OD_600_ value of 1.5. Bacterial cells were harvested, and total RNA was extracted using TRIzol Reagent. cDNA was generated from 1 μg total RNA using a FastKing First Strand cDNA Synthesis Kit (Tiangen Biotech, China). The relative mRNA level of each target gene was determined using the classical 2^−ΔΔCt^ method with the 16S rRNA gene as the internal control. Primers used in this work were synthesized by GENEWIZ Biotechnology Co. Ltd. (Suzhou, China). Primers used in this work (Table 1) were synthesized by GENEWIZ Biotechnology Co., Ltd. (Suzhou, China) based on the solid phase subphosphorylation, and purified using the C18 column desalt method.

**Table 1:** Primers used for qPCR in this study.

Target	Primers (forward/reverse, 5′-3′)	Size (bp)	References
VP0228	GCTGCTAGTTTCTTATGTTC/GTCCGATCAAACCAACAAGG	191	This study
VP0775	ACAAGGCACTAGGCATCC/GACCATCTGTTCGGCTAAG	180	Lu et al. (2021)
VP0778	CTCGTGCGGAATTTGCTGATG/GGTTGTTGGTGTAAATGCTTG	132	Lu et al. (2021)
VP1393	GGTCAACCTACTGGTCAACG/TAGTGCTCTTGCTTGCCTTG	161	Zhang et al. (2017)
VP1467	TTGGTATCAAAGCAGAGCACTC/CTAAGGACATCCATTGGCAAGG	174	This study
VP1667	GGAATGGATTGGAATCGTC/CCACCGTCTTTTATTTTGC	175	Zhang et al. (2019)
VP1687	TGCTCACCGTTGCCAAATAG/GCGACGCTTTCATGTATTGC	113	Zhang et al. (2019)
VP1698	AAGAGGAGCACGATATGAG/AACTGTCCACCACACTTC	152	Zhang et al. (2016)
VP1768	TGAATCTCAGGCCAACATGC/AAATTCGCCGTTATTGATACCG	199	This study
VP2111	GTTATGTCGCTACTCCGCAGC/CGAAAACACCGCATCACCAAA	107	This study
VP2362	ACCTAGCGTCAGACAAAGGC/TGAACTGGACCGAAAGACAGG	118	This study
VP2467	TTACAACCAAGACGGCAC/CCTTCCCAGAAACCAACA	159	Zhang et al. (2023a)
VP2523	GGCTGAGCTGCATTACCAAG/TCCCACCGTCGATAGAACTG	167	Zhang et al. (2023b)
VP2700	AGCGTTGATGAATAAAGGGA/GAACAACTGACGAGAAAACA	139	This study
VPA0168	GGTATCTGCGTCATCACTTC/TGCAAATAGCGCCACCAAAG	163	This study
VPA0202	GCTTTACAACAACTACGTGG/GGTATCTGACAAAGTATCAC	118	This study
VPA0299	TGCCAAACGACTGAACATGA/GAGGTAAGTTGTCTGCCAGT	146	This study
VPA0360	TCGTTCTTTACCTACGCCTTA/TGCCAATAACACTCGATAGAGC	176	This study
VPA0556	TGAAGCGGAATTTGTGCGTG/ATCTGGTGTTGTCGCCATGT	154	This study
VPA0602	TATCACAGCAACAGCAAACC/GTTTGGCACGTATGGAGATT	152	This study
VPA0609	GCACAGAACTTATCGAAAGCC/ATCAAAAGATCATTCGAGATCGC	133	This study
VPA0717	CTATATTTAACCCAACCAGCC/GATCGAAGACTTCAGCCCCTA	76	This study
VPA1027	TAAAGGTGAAGCGACAGCG/AATCATATAGGCGTGTTGC	137	Qiu et al. (2020)
VPA1191	TCAGGATGTCACGGTAATCG/GCCTGAAATCTGTGCTGTGA	97	This study
VPA1346	GCTGCTGCGAATGATATTGC/TCTGGCGGTTGTATCCTCTG	150	This study
VPA1370	AATCCGCCAAGGTGTAAAGC/GTTGACGCTGATGGTAGTGG	171	This study
VPA1446	GCCTGAAATCCTAATGCTC/AGTGTCAGAAGGTGTATCAAC	181	Zhang et al. (2021)
VPA1418	GATTTAGTCGGCAACAACAC/ATCCCAGTTGTTTGTCGAGC	123	This study
VPA1683	AGACCACTACGAAGAGCTAC/GTACTGGATCTTGCCGATTG	118	This study
*cpsA*	GAGAGCGGCAACCTATATCG/CGCCACGCCAACAGTAATG	194	Zhang et al. (2023b)
*cpsE*	GTCTCTTGGCGTGCTTATC/GAGCCGACTTTACCCATTTG	154	Zhang et al. (2023b)
16S rRNA	GACACGGTCCAGACTCCTAC/GGTGCTTCTTCTGTCGCTAAC	179	Zhang et al. (2023b)

### Experimental replicates and statistical methods

The growth curves, CV staining, c-di-GMP quantification, swarming, swimming, cytotoxicity and adhesion assays were performed at least three independent times with three biological replicates in each time, and the results were expressed as the mean ± standard deviation (SD). Paired Student’s *t*-tests were used to calculate statistical the significance with a *p*-value less than 0.05 considered significant. Colony morphology and OP-TR colony switching assays were performed at least three independent times.

## Results

### Chloramphenicol inhibited the growth of *Vibrio parahaemolyticus*

**Figure 1:**
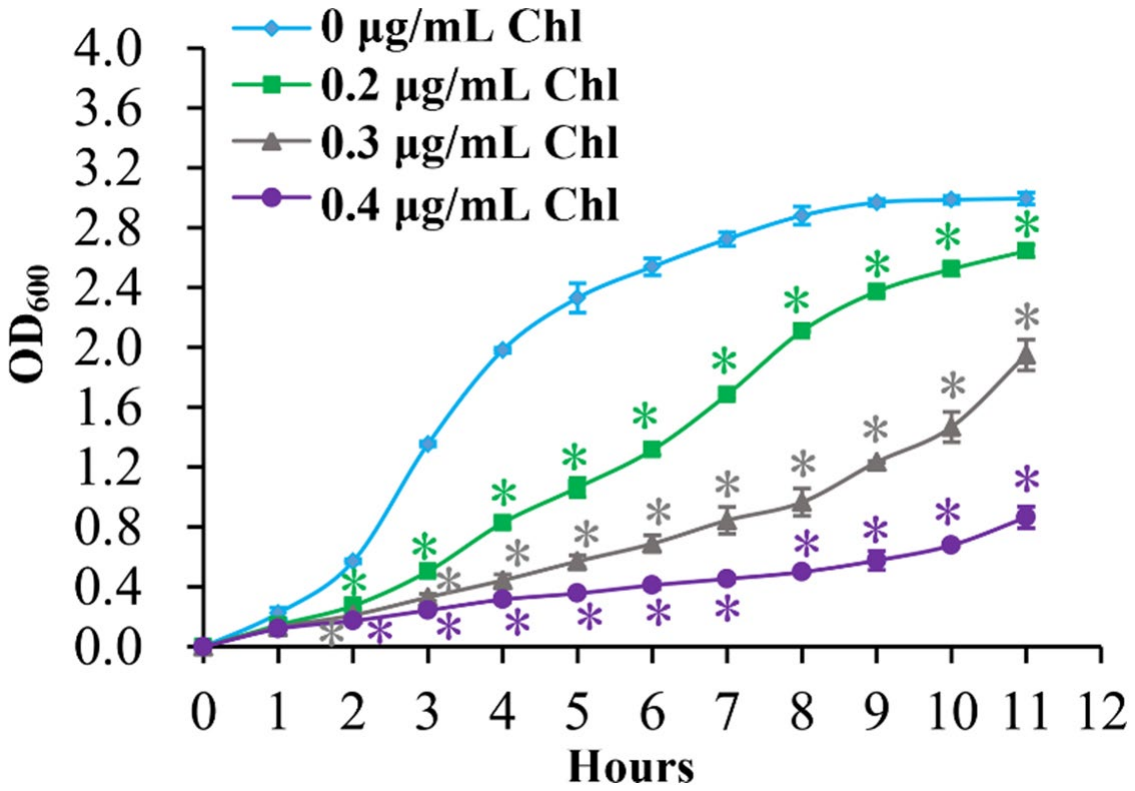
Growth curves of *V. parahaemolyticus*. *V. parahaemolyticus* was grown in HI broth supplemented with different concentrations of chloramphenicol at 37°C with shaking at 200 rpm, and the OD_600_ values were monitored at 1 h intervals. Experiments were performed two times with three replicates per trial for each condition. Chl indicates chloramphenicol. The asterisk (*) represents *p* < 0.01.

The growth curves of *V. parahaemolyticus* in HI broth supplemented with 0, 0.2, 0.3, and 0.4 μg/mL chloramphenicol were measured to investigate whether sublethal doses of chloramphenicol have some effects on the growth of the bacterium. As shown in Figure 1, the growth rates of *V. parahaemolyticus* in the presence of chloramphenicol were significantly slower than in the absence of chloramphenicol, and *V. parahaemolyticus* manifested slower and slower growth rates with the increasing of chloramphenicol. These results suggested that sublethal doses of chloramphenicol were able to inhibit the growth of *V. parahaemolyticus*.

### Sublethal doses of chloramphenicol reduced the biofilm formation capacity of *Vibrio parahaemolyticus*

**Figure 2:**
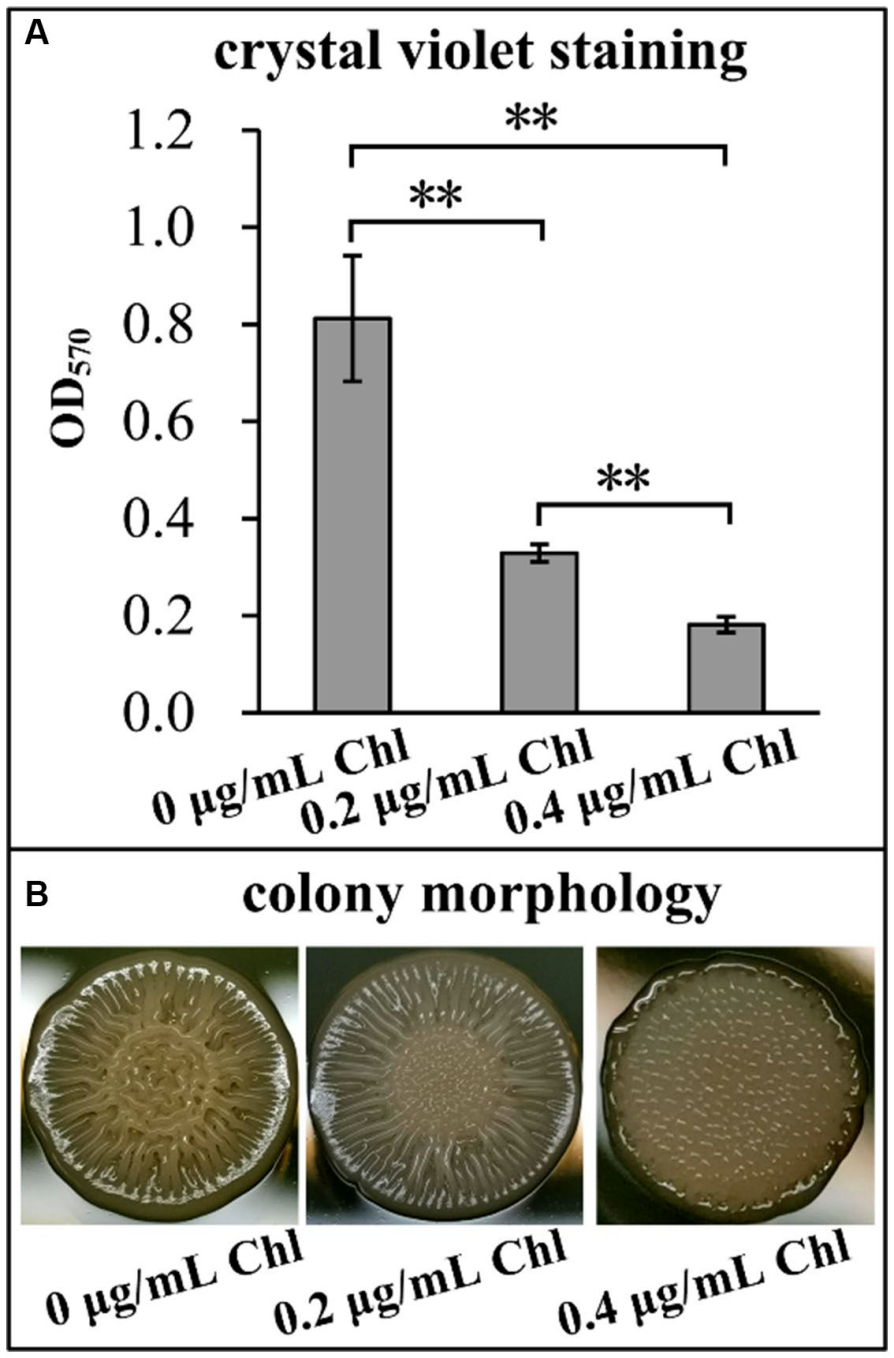
Sublethal doses of chloramphenicol reduced the biofilm formation capacity of *V. parahaemolyticus*. Biofilm formation ability of *V. parahaemolyticus* was assessed by intensity of crystal violet staining **(A)** and rugose colony morphology **(B)**. Pictures were representative of three independent experiments with three replicates each. The double asterisk (**) represents *p* < 0.01. Chl indicates chloramphenicol.

The effects of sublethal doses of chloramphenicol on the biofilm formation capacity of *V. parahaemolyticus* were assessed using the CV staining and colony morphology assays. As shown in Figure 2A, *V. parahaemolyticus* grown in the presence of sublethal doses of chloramphenicol exhibited significantly less normalized CV staining relative to that grown in the absence of chloramphenicol (*p* < 0.05), and the higher chloramphenicol concentration was added, the less normalized CV staining was detected. As further confirmed by the colony morphology (Figure 2B), the colonies of *V. parahaemolyticus* on the HI plate supplemented with 0.4 μg/mL of chloramphenicol were smooth, whereas those on other concentrations of chloramphenicol containing plates were wrinkly. However, the colonies of *V. parahaemolyticus* on the HI plate without chloramphenicol were much more wrinkled than those on the plate supplemented with 0.2 μg/mL of chloramphenicol (Figure 2B). These results suggested that chloramphenicol dose-dependently inhibited the biofilm formation capacity of *V. parahaemolyticus*.

### Chloramphenicol did not affect the OP-TR colony variation of *Vibrio parahaemolyticus*

**Figure 3:**
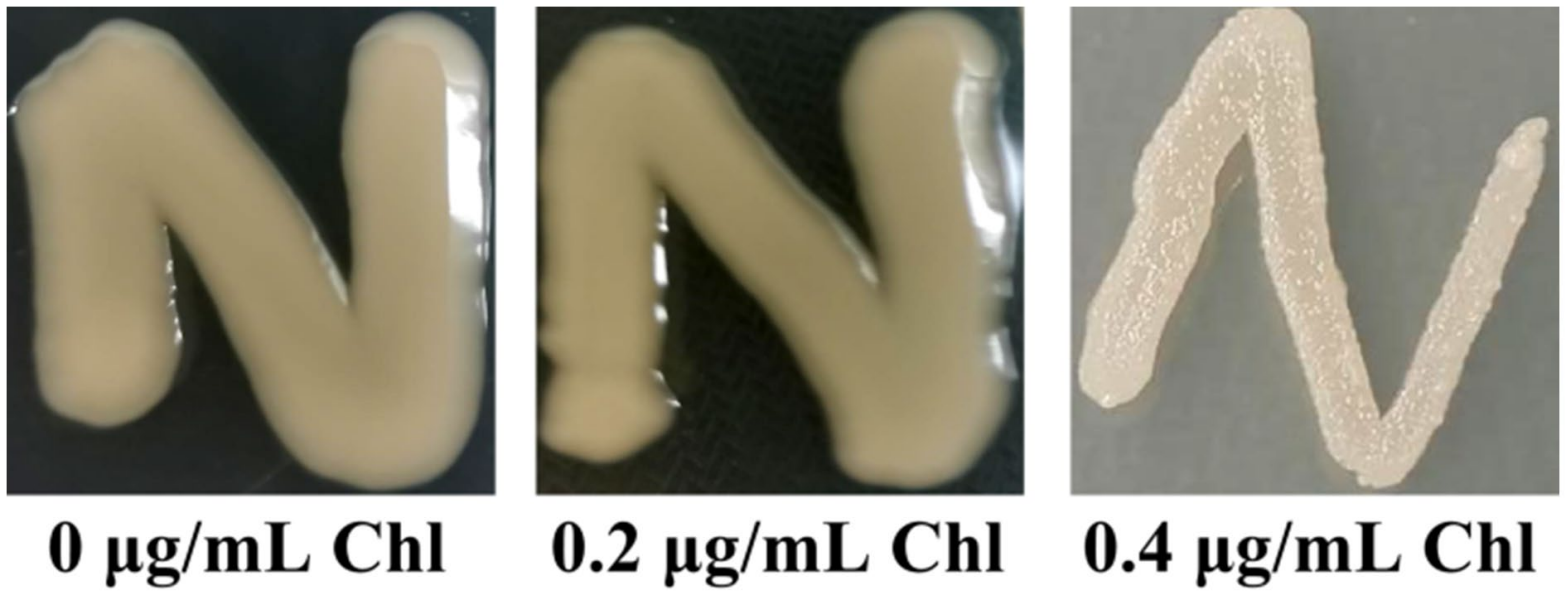
The OP-TR colony variation of *V. parahaemolyticus*. A small amount of *V. parahaemolyticus* cells was streaked onto a HI plate containing 0, 0.2 or 0.4 μg/mL chloramphenicol, and then statically incubated at 37°C for 24 h. Pictures were representative of two independent experiments with three replicates each. Chl indicates chloramphenicol.

*Vibrio parahaemolyticus* naturally switches between OR and TP colony phenotypes based on whether capsular polysaccharide (CPS) production or not (Chen et al., 2010). In this study, the data showed that *V. parahaemolyticus* manifested the OP colony phenotype no matter whether the chloramphenicol presence or not (Figure 3), suggesting that sublethal doses of chloramphenicol did not affect the OP-TR colony variation of *V. parahaemolyticus*. However, the lawn of *V. parahaemolyticus* on the HI plate supplemented with 0.4 μg/mL of chloramphenicol seemed to be much drier than those under the other two conditions (Figure 3), which may be related to the growth inhibition of chloramphenicol (Figure 1).

### Sublethal doses of chloramphenicol reduced the motor capacity of *Vibrio parahaemolyticus*

**Figure 4:**
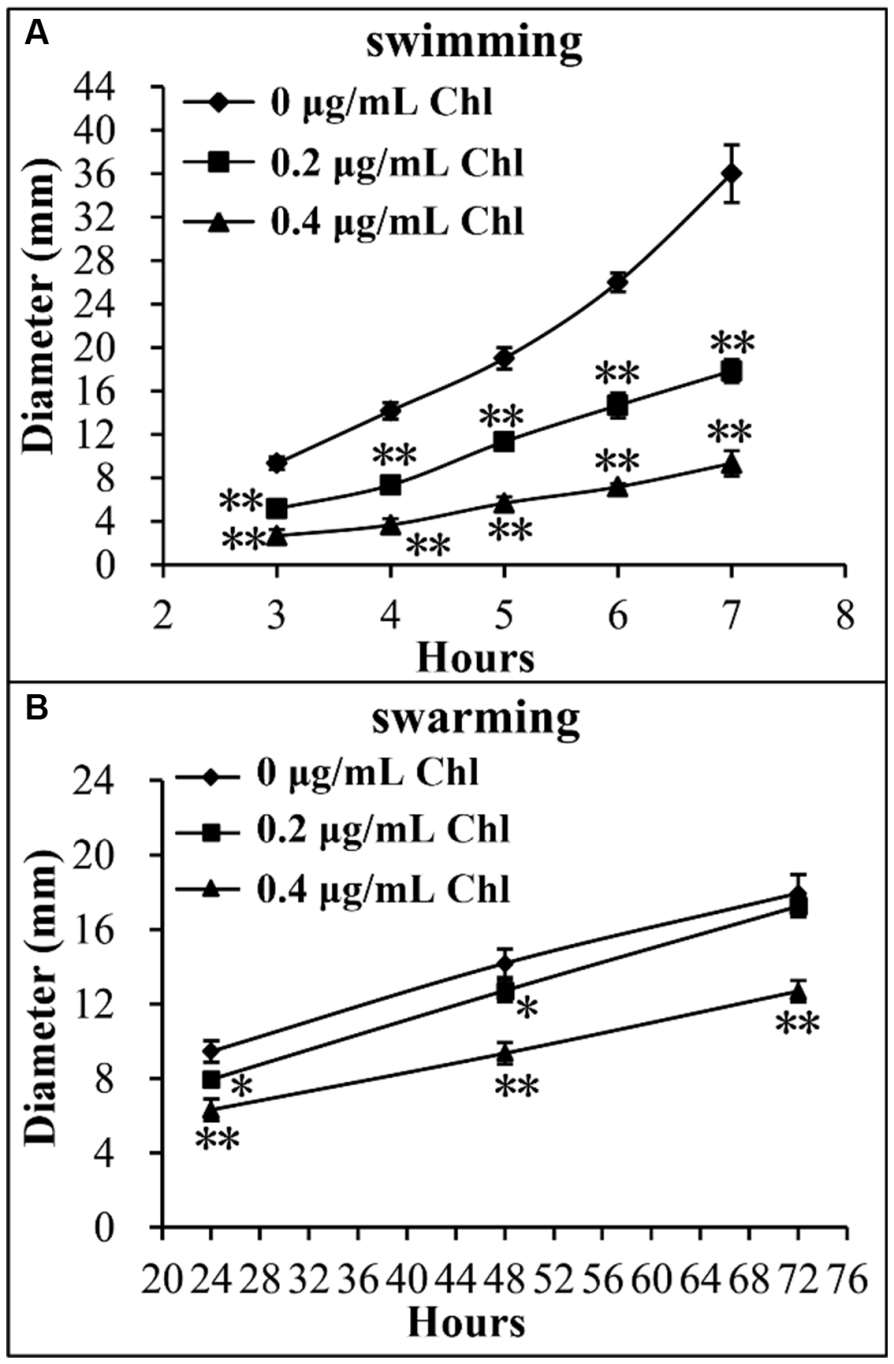
Sublethal doses of chloramphenicol inhibited the motility of *V. parahaemolyticus*. Swimming **(A)** or swarming **(B)** capacity of *V. parahaemolyticus* was measured by detection of the diameters of swimming or swarming areas in a semi-solid swimming or on swarming agar. The data at each time point are expressed as the mean ± SD of three independent experiments with three replicates each. The single asterisk (*) represents *p* < 0.05, whereas the double asterisk (**) indicates *p* < 0.01. Chl indicates chloramphenicol.

Effect of sublethal doses of chloramphenicol on the motor capacity of *V. parahaemolyticus* was investigated using the swimming and swarming assays. As shown in Figure 4, the swimming and swarming motility of *V. parahaemolyticus* were significantly decreased in the presence of sublethal doses of chloramphenicol relative to the conditions without chloramphenicol at all time points tested. Higher concentration of chloramphenicol was able to reduce more the motor ability of *V. parahaemolyticus*. These results suggested that chloramphenicol dose-dependently inhibited swimming and swarming motility of *V. parahaemolyticus*.

### Sublethal doses of chloramphenicol inhibited the production of intracellular c-di-GMP

**Figure 5:**
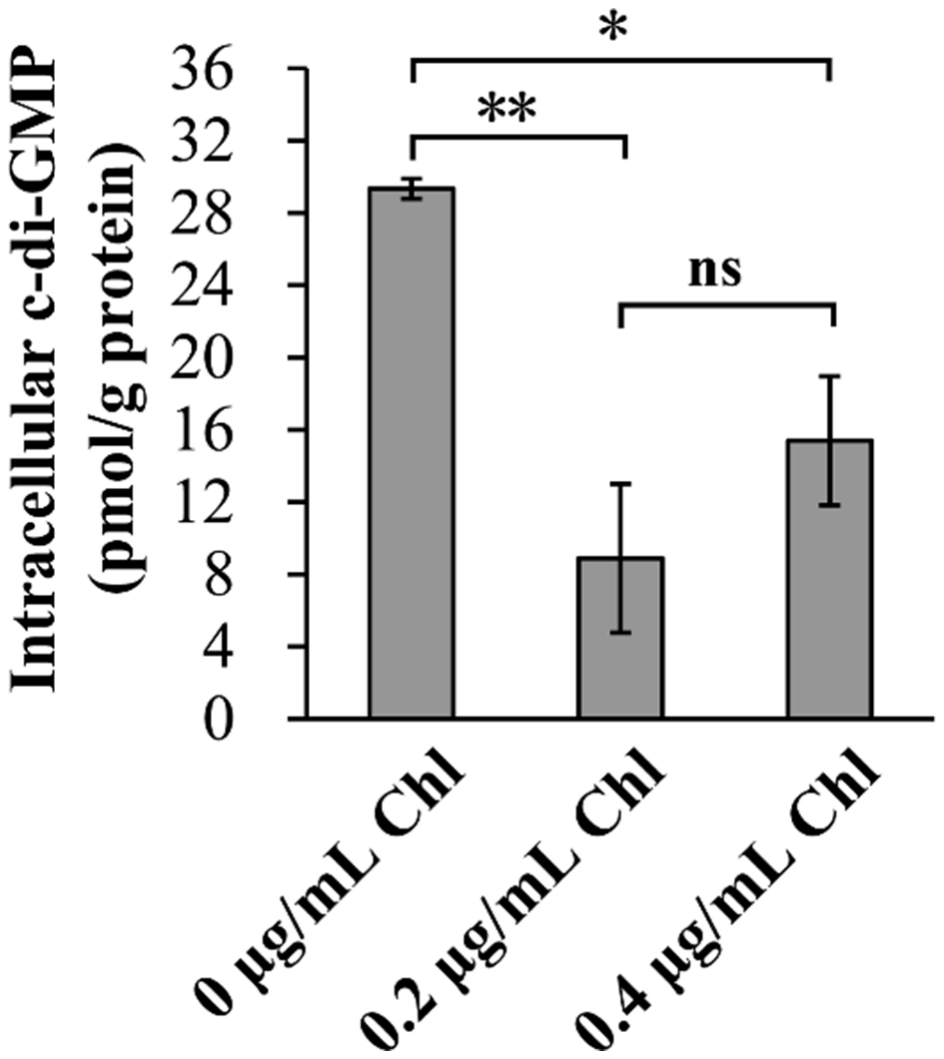
Sublethal doses of chloramphenicol reduced the intracellular c-di-GMP levels of *V. parahaemolyticus*. Bacterial cells were cultured 37°C with shaking at 200 rpm in HI broth containing 0, 0.2 or 0.4 μg/mL chloramphenicol, and then harvested at an OD_600_ value of 1.5. The data are expressed as the mean ± SD of three independent experiments with three replicates each. The single (*) and double (**) asterisks represent *p* < 0.05 and *p* < 0.01, respectively, whereas the ‘ns’ indicates *p* > 0.05. Chl indicates chloramphenicol.

The intracellular c-di-GMP levels were investigated to assess whether sublethal doses of chloramphenicol affect the c-di-GMP pool of *V. parahaemolyticus*. As shown in Figure 5, the intracellular c-di-GMP levels of *V. parahaemolyticus* were remarkably reduced in the presence of sublethal doses of chloramphenicol, suggesting that sublethal doses of chloramphenicol was able to inhibit the synthesis of intracellular c-di-GMP in *V. parahaemolyticus*.

### Sublethal doses of chloramphenicol reduced the adherence and cytotoxicity of *Vibrio parahaemolyticus*

**Figure 6:**
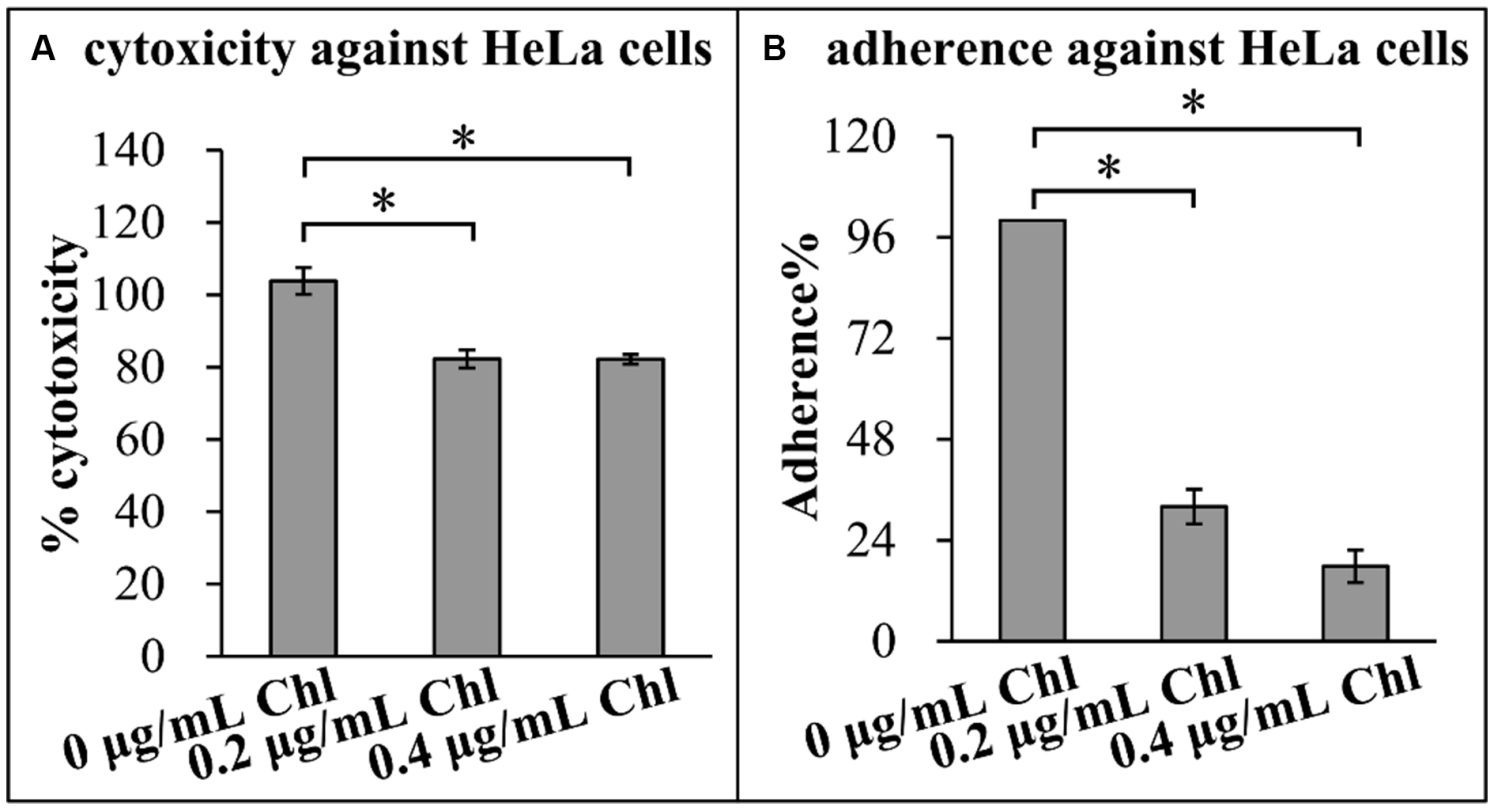
Sublethal doses of chloramphenicol reduced the virulence of *V. parahaemolyticus*. The results were expressed as the mean ± SD from at least two independent experiments with four replicates. The single asterisk (*) represents *p* < 0.01. Chl indicates chloramphenicol. **(A)** Cytotoxicity against HeLa cells. The cytotoxicity of *V. parahaemolyticus* against HeLa cells was evaluated in terms the release of LDH. **(B)** Adherence against HeLa cells. HeLa cells were infected with *V. parahaemolyticus* cells at a MOI of 10. The percent adherence was calculated as bacterial cells adhered/input bacterial cells. The adherence of *V. parahaemolyticus* cultured in the absence of chloramphenicol condition was normalized to 100%.

Effect of sublethal doses of chloramphenicol on the adherence and cytotoxicity of *V. parahaemolyticus* was investigated using the following virulence-associated phenotypes. First, cytotoxicity against HeLa cells of *V. parahaemolyticus* grown in the presence or absence of chloramphenicol was investigated regarding to the release of LDH from cultured cells (Figure 6A). The cytotoxicity of *V. parahaemolyticus* grown in the conditions with chloramphenicol was significantly reduced relative to that grown in the condition without chloramphenicol, suggesting that chloramphenicol inhibited the cytotoxicity of *V. parahaemolyticus* against HeLa cells. Second, adhesion activity of *V. parahaemolyticus* to HeLa cells was investigated using the adhesion assay (Figure 6B). The results showed that sublethal doses of chloramphenicol were able to influence the adhesion of *V. parahaemolyticus* to HeLa cells in a dose-dependent manner, as that the adhesion rates of *V. parahaemolyticus* under the conditions of 0.2 and 0.4 μg/mL chloramphenicol were only approximately 30 and 20% of that under the condition without chloramphenicol, respectively. Taken together, these results suggested that sublethal doses of chloramphenicol inhibited the adherence and cytotoxicity of *V. parahaemolyticus*.

### Sublethal dose of chloramphenicol affected global gene expression of *Vibrio parahaemolyticus*

**Figure 7:**
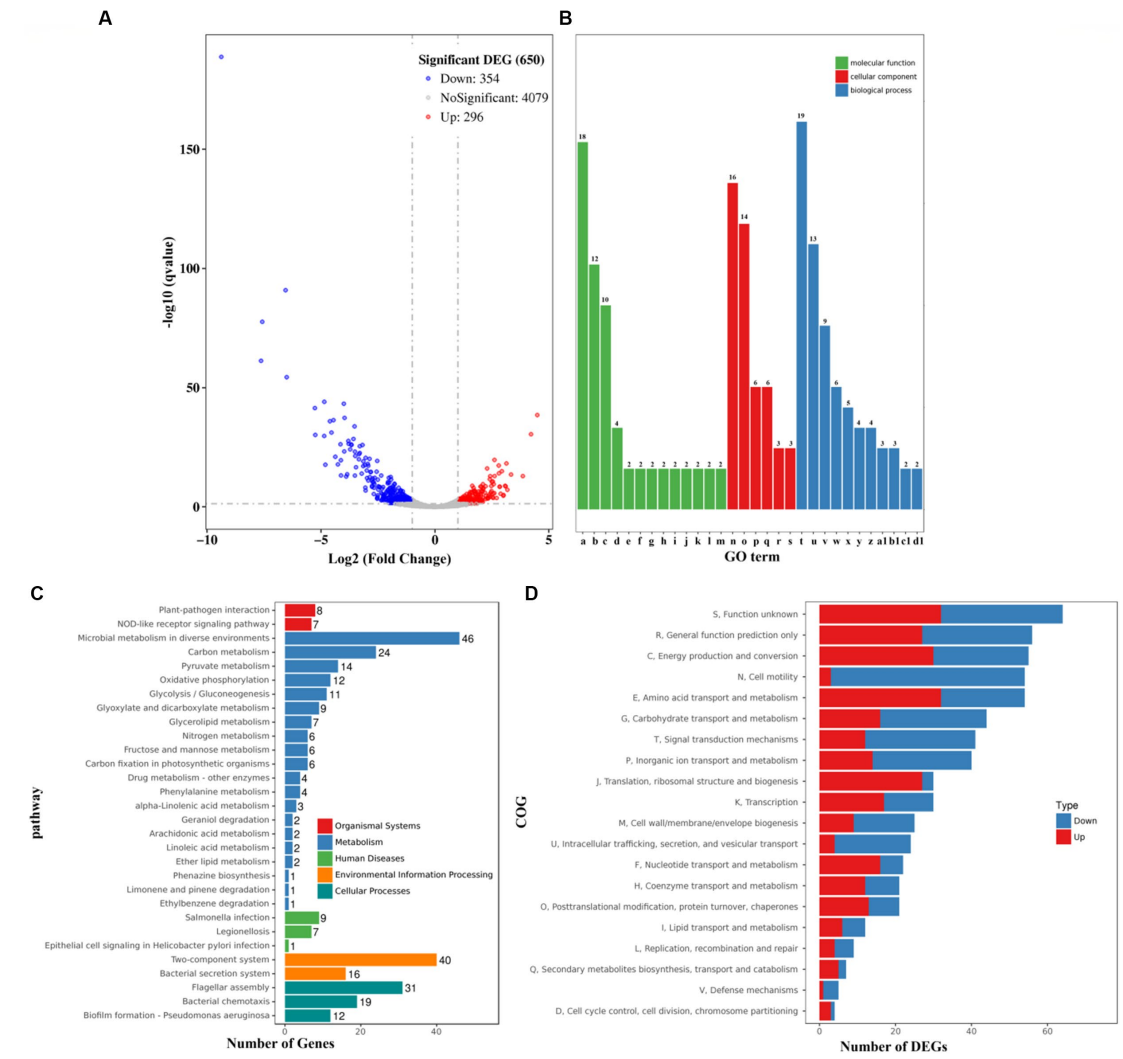
Expression profiles of *V. parahaemolyticus* in the presence of 0.2 μg/mL chloramphenicol. **(A)** Volcano plot. Red, blue and gray points represent the up-regulated, down-regulated and no-significant genes, respectively. **(B)** The enrichment of gene ontology (GO) term. Green, red and blue bars represent molecular function, cellular component and biological process, respectively. The number on the top of each bar indicates the number of enriched genes. Alphabets from a-d1 in the horizontal axis represent structural constituent of ribosome, rRNA binding, structural molecule activity, cytochrome-c oxidase activity, AMP binding, oxo-acid-lyase activity, phosphoenolpyruvate-glycerone phosphotransferase activity, glycerone kinase activity, cytochrome o ubiquinol oxidase activity, pyrimidine-nucleoside phosphorylase activity, thymidine phosphorylase activity, guanosine phosphorylase activity, uridine phosphorylase activity, ribosome, extracellular region, membrane, bacterial-type flagellum filament, type III protein secretion system complex, proton-transporting ATP synthase complex/coupling factor F (o), translation, bacterial-type flagellum-dependent cell motility, bacterial-type flagellum organization, ‘*de novo*’ IMP biosynthetic process, glycerol metabolic process, bacterial-type flagellum assembly, glycerol catabolic process, protein secretion by the type III secretion system, lipid catabolic process, propionate metabolic process/methylcitrate cycle, and L-threonine catabolic process to glycine, respectively. **(C)** Pathways of differentially expressed genes were enriched by Kyoto Encyclopedia of Genes and Genomes (KEGG). The vertical axis represents KEGG classification, while the horizontal axis represents the number of DEGs. **(D)** Cluster of Orthologous Groups of proteins (COG). The vertical axis represents COG classification, whereas the horizontal axis represents the number of DEGs.

The growth of *V. parahaemolyticus* was severely inhibited by 0.4 μg/mL chloramphenicol (Figure 1). Therefore, the gene expression profiles of *V. parahaemolyticus* grown in HI broth supplemented with 0.2 (test) chloramphenicol were compared with those grown in HI broth containing 0 μg/mL chloramphenicol (reference) by RNA-seq to investigate the cellular pathways involved in the response to the sublethal dose of chloramphenicol. As shown in Figure 7A, a total of 650 genes were significantly differentially expressed in the response to chloramphenicol stress, of these 354 were down-regulated and 296 were up-regulated. The enrichment of gene ontology (GO) term showed that the DEGs were involved in molecular function (13 GO terms, 62 DEGs), followed by biological process (11 GO terms, 70 DEGs) and cellular component (6 GO terms, 48 DEGs) (Figure 7B). The results of Kyoto Encyclopedia of Genes and Genomes (KEGG) analysis showed that 15, 163, 17, 56 and 62 DEGs were involved in organismal systems, metabolism, human diseases, environmental information processing and cellular processes, respectively (Figure 7C). The results of Cluster of Orthologous Groups of proteins (COG) enrichment showed that the DEGs were divided into 20 functional categories, and the 5 top pathways were function unknown, general function prediction only, energy production and conversion, cell motility and amino acid transport and metabolism (Figure 7D). The detailed information of DEGs was listed in Supplementary Table S1.

### Selected DEGs of interest

A total of 6 genes associated with antibiotic resistance were significantly differentially expressed in the response of chloramphenicol (Table 2), of these 3 were downregulated (VP2167, VPA0520, and VPA1590) and 5 were upregulated (VP3019, VPA0168, VPA1190, VPA1191, and VPA1647). Five genes encoding GGDEF and/or EAL-domain proteins were significantly differentially expressed, including 2 downregulated genes (VP1768 and VPA0202) and 3 upregulated genes (VPA0360, VPA0556, and VPA0609). Additionally, 1 lateral flagellar gene (VPA1546), 43 polar flagellar genes, 1 CPS-related gene (VP0228), 6 *scv* genes (VP1458, VP1461, VP1463, VP1464, VP1466, and VP1467), and 5 MSHA genes (VP2699, VP2700, VP2701, VP2702, and VP2703), 28 T3SS1-associated genes including *exsD*, 5 T3SS2-associated genes (VPA1342, VPA1345, VPA1346, VPA1361, and VPA1370), 1 T6SS1-related gene (*hcp1*), and 13 T6SS2-related genes were remarkably downregulated in the presence of chloramphenicol (Table 2). In addition, at least 23 putative regulatory genes were significantly differentially expressed including 11 downregulated and 12 upregulated genes (Table 2). The DEGs also contained other of interest genes including 2 antioxidative genes (*katE1* and *ahpC1*) and 12 outer membrane protein (OMP) encoding genes (Table 2).

**Table 2:** Selected genes from the DEGs.

Locus_tag	Gene name	FoldChange	Regulation	Production
c-di-GMP				
VP1768		0.4828	Down	EAL domain-containing protein
VPA0202		0.4460	Down	GGDEF domain-containing protein
VPA0360		3.7684	Up	GGDEF domain-containing protein
VPA0556		2.8669	Up	Sensor domain-containing diguanylate cyclase
VPA0609		2.0915	Up	Bifunctional diguanylate cyclase/phosphodiesterase
Lateral flagella				
VPA0261		2.5467	Up	Flagellar export chaperone FlgN
VPA1546	*flhA*	0.4280	Down	Flagellar biosynthesis protein FlhA
Polar flagellum				
VP0771	*flgM*	0.4615	Down	Flagellar biosynthesis anti-sigma factor FlgM
VP0775	*flgB*	0.2595	Down	Flagellar basal body rod protein FlgB
VP0776	*flgC*	0.1843	Down	Flagellar basal body rod protein FlgC
VP0777	*flgD*	0.2071	Down	Flagellar hook assembly protein FlgD
VP0778	*flgE*	0.1785	Down	Flagellar hook protein FlgE
VP0780	*flgF*	0.0626	Down	Flagellar basal body rod protein FlgF
VP0781	*flgG*	0.0759	Down	Flagellar basal-body rod protein FlgG
VP0782	*flgH*	0.1017	Down	Flagellar basal body L-ring protein FlgH
VP0783	*flgI*	0.1317	Down	Flagellar basal body P-ring protein FlgI
VP0784	*flgJ*	0.1504	Down	Flagellar assembly peptidoglycan hydrolase FlgJ
VP0785	*flgK*	0.0635	Down	Flagellar hook-associated protein FlgK
VP0786	*flgL*	0.0996	Down	Flagellar hook-associated protein FlgL
VP0788	*flaC*	0.0887	Down	Flagellin
VP0790	*flaD*	0.0345	Down	Flagellin
VP0791	*flaE*	0.2255	Down	Flagellin
VP2111	*motY*	0.1216	Down	OmpA family protein
VP2224	*orf3*	0.4790	Down	DUF2802 domain-containing protein
VP2226	*orf2*	0.2940	Down	Chemotaxis protein CheW
VP2227	*orf1*	0.3686	Down	ParA family protein
VP2228	*cheB*	0.3924	Down	Chemotaxis response regulator protein-glutamate methylesterase
VP2229	*cheA*	0.2926	Down	Chemotaxis protein CheA
VP2230	*cheZ*	0.3762	Down	Protein phosphatase CheZ
VP2231	*cheY*	0.4371	Down	Chemotaxis response regulator CheY
VP2232	*fliA*	0.3984	Down	RNA polymerase sigma factor FliA
VP2235	*flhA*	0.4259	Down	Flagellar biosynthesis protein FlhA
VP2236	*flhB*	0.4912	Down	Flagellar biosynthesis protein FlhB
VP2237	*fliR*	0.2783	Down	Flagellar type III secretion system protein FliR
VP2239	*fliP*	0.3496	Down	Flagellar type III secretion system pore protein FliP
VP2242	*fliM*	0.3481	Down	Flagellar motor switch protein FliM
VP2243	*fliL*	0.3906	Down	Flagellar basal body-associated protein FliL
VP2244	*fliK*	0.2561	Down	Flagellar hook-length control protein FliK
VP2245	*fliJ*	0.3063	Down	Flagella biosynthesis chaperone FliJ
VP2246	*fliI*	0.4777	Down	Flagellar protein export ATPase FliI
VP2247	*fliH*	0.4270	Down	Flagellar assembly protein FliH
VP2252	*flaL*	0.4478	Down	PAS domain-containing protein
VP2254	*fliS*	0.0792	Down	Flagellar export chaperone FliS
VP2255	*flaI*	0.0573	Down	Flagellar protein FliT
VP2256	*fliD*	0.0878	Down	Flagellar filament capping protein FliD
VP2257	*flaG*	0.0784	Down	Flagellar protein FlaG
VP2258	*flaA*	0.0673	Down	Flagellin
VP2259	*flaB*	0.0483	Down	Flagellin
VP2261	*flaF*	0.0411	Down	Flagellin
VP2811	*motX*	0.1480	Down	Sel1 repeat family protein
Capsule polysaccharide				
VP0228		0.4513	Down	Oligosaccharide repeat unit polymerase
Scv exopolysaccharide				
VP1458	*scvO*	0.2906	Down	Sugar transferase
VP1461	*scvM*	0.3120	Down	Glycosyltransferase
VP1463	*scvK*	0.2733	Down	Glycosyltransferase
VP1464	*scvJ*	0.1752	Down	O-antigen ligase family protein
VP1466	*scvH*	0.3166	Down	Glycosyltransferase family 4 protein
VP1467	*scvG*	0.2408	Down	Glycosyltransferase
Type IV pilin				
VP2523	*pilA*	3.4930	Up	Pilin
VP2699	*mshF*	0.3401	Down	MSHA biogenesis protein MshF
VP2700	*mshG*	0.4121	Down	Type II secretion system F family protein
VP2701	*mshE*	0.3945	Down	GspE/PulE family protein
VP2702	*mshN*	0.4795	Down	MSHA biogenesis protein MshN
VP2703	*mshM*	0.4555	Down	ExeA family protein
T3SS1				
VP1656	*vopD*	0.2107	Down	Type III secretion system translocon subunit VopD
VP1657	*vopB*	0.1130	Down	Type III secretion system translocon subunit VopB
VP1658	*vcrH*	0.3639	Down	SycD/LcrH family type III secretion system chaperone VcrH
VP1659	*vcrV*	0.2281	Down	Type III secretion system needle tip protein VcrV
VP1660	*vcrG*	0.2617	Down	LcrG family type III secretion system chaperone VcrG
VP1661	*vcrR*	0.3482	Down	LcrR family type III secretion system chaperone VcrR
VP1662	*vcrD*	0.3107	Down	SctV family type III secretion system export apparatus subunit VcrD
VP1664	*vscX*	0.3616	Down	Type III secretion system protein VscX
VP1666		0.2139	Down	TyeA family type III secretion system gatekeeper subunit
VP1667	*vopN*	0.2554	Down	SctW family type III secretion system gatekeeper subunit VopN
VP1668	*vscN*	0.3004	Down	SctN family type III secretion system ATPase VscN
VP1669	*vscO*	0.2882	Down	Type III secretion system central stalk protein VscO
VP1670	*vscP*	0.3062	Down	Type III secretion system needle length determinant VscP
VP1671	*vscQ*	0.1864	Down	SctQ family type III secretion system cytoplasmic ring protein VscQ
VP1672	*vscR*	0.2178	Down	SctR family type III secretion system export apparatus subunit VscR
VP1680	*vopQ*	0.1078	Down	Type III secretion system effector VopQ
VP1682	*vecA*	0.0877	Down	CesT family type III secretion system chaperone VecA
VP1686	*vopS*	0.2719	Down	T3SS effector adenosine monophosphate-protein transferase VopS
VP1687		0.3800	Down	CesT family type III secretion system chaperone
VP1689	*vscK*	0.2189	Down	SctK family type III secretion system sorting platform protein VscK
VP1690	*vscJ*	0.3265	Down	SctJ family type III secretion inner membrane ring lipoprotein Vsc
VP1692	*vscH*	0.2758	Down	YopR family T3SS polymerization control protein VscH
VP1693	*vscG*	0.3504	Down	YscG family type III secretion system chaperone VscG
VP1694	*vscF*	0.2839	Down	Type III secretion system needle filament protein VscF
VP1695	*vscD*	0.2477	Down	SctD family type III secretion system inner membrane ring subunit VscD
VP1696	*vscC*	0.2480	Down	SctC family type III secretion system outer membrane ring subunit VscC
VP1697	*vscB*	0.2263	Down	YscB family type III secretion system chaperone VscB
VP1698	*esxD*	0.4088	Down	Type III secretion system regulon anti-activator ExsD
T3SS2				
VPA1343		0.3765	Down	Hypothetical protein
VPA1345		0.4713	Down	Hypothetical protein
VPA1346	*vopA*	0.3708	Down	Type III secretion system YopJ family effector VopA
VPA1361	*vopD2*	0.3781	Down	Type III secretion system translocator protein VopD2
VPA1370	*vopL*	0.4842	Down	Type III secretion system effector VopL
T6SS1				
VP1393	*hcp1*	0.4922	Down	Hcp family type VI secretion system effector
T6SS2				
VPA1027	*hcp2*	0.1952	Down	Type VI secretion system tube protein Hcp
VPA1028	*tssH*	0.2916	Down	Type VI secretion system ATPase TssH
VPA1029	*tssG*	0.3176	Down	Type VI secretion system baseplate subunit TssG
VPA1030	*tssF*	0.2643	Down	Type VI secretion system baseplate subunit TssF
VPA1031	*tssE*	0.2103	Down	Type VI secretion system baseplate subunit TssE
VPA1032		0.1504	Down	Protein of avirulence locus
VPA1033	*tssC*	0.2006	Down	Type VI secretion system contractile sheath large subunit
VPA1034	*tssC*	0.1369	Down	Type VI secretion system contractile sheath large subunit
VPA1035	*tssB*	0.1730	Down	Type VI secretion system contractile sheath small subunit
VPA1036	*tssA*	0.2603	Down	Type VI secretion system protein TssA
VPA1037		0.2925	Down	Protein phosphatase 2C domain-containing protein
VPA1038	*tagF*	0.2788	Down	Type VI secretion system-associated protein TagF
VPA1039	*tssM*	0.3321	Down	Type VI secretion system membrane subunit TssM
Putative regulators				
VP0243		2.0226	Up	Transcriptional regulator
VP0247	*rraA*	0.4376	Down	Ribonuclease E activity regulator RraA
VP0624		3.1754	Up	LysR family transcriptional regulator
VP1229		4.0701	Up	TetR/AcrR family transcriptional regulator
VP1376		0.2983	Down	Response regulator
VP1649		3.1477	Up	GntR family transcriptional regulator
VP1962		0.4521	Down	Crp/Fnr family transcriptional regulator
VP2009		0.4655	Down	Response regulator transcription factor
VP2396		2.2575	Up	LacI family DNA-binding transcriptional regulator
VP2450		2.0423	Up	MarR family transcriptional regulator
VP2632		0.4270	Down	LacI family DNA-binding transcriptional regulator
VP2777		0.4493	Down	Transcriptional regulator
VPA0011		2.4106	Up	YebC/PmpR family DNA-binding transcriptional regulator
VPA0053		0.4913	Down	TetR/AcrR family transcriptional regulator
VPA0299		0.3864	Down	LysR family transcriptional regulator
VPA0358		2.3513	Up	Helix-turn-helix transcriptional regulator
VPA0359		4.0065	Up	Helix-turn-helix transcriptional regulator
VPA0602		0.3564	Down	LysR family transcriptional regulator
VPA0717		3.4322	Up	LysR family transcriptional regulator
VPA0804		0.4881	Down	XRE family transcriptional regulator
VPA1178		2.7744	Up	Sugar-binding transcriptional regulator
VPA1446	*cpsQ*	3.9098	Up	Helix-turn-helix transcriptional regulator
VPA1636		0.3543	Down	Helix-turn-helix transcriptional regulator
Outer membrane protein				
VP0760	*chiP*	0.2340	Down	Chitoporin
VP0887		0.4823	Down	Outer membrane lipoprotein carrier protein LolA
VP1008		2.4345	Up	Porin
VP1218		2.2271	Up	MtrB/PioB family decaheme-associated outer membrane protein
VP1356		2.8495	Up	BamA/TamA family outer membrane protein
VP2176	*aqpZ*	2.8829	Up	Aquaporin Z
VP2362	*ompK*	0.3768	Down	Outer membrane protein OmpK
VP2385		0.0051	Down	Aquaporin
VP2467	*ompU*	0.1193	Down	Porin
VPA0242		0.3722	Down	Porin family protein
VPA0527	*ompN*	2.6928	Up	Porin
VPA0860		4.9090	Up	Outer membrane protein transport protein
Antibiotic resistance				
VP3019		2.3726	Up	Multidrug effflux MFS transporter
VPA0168	*emrD*	2.1828	Up	Multidrug efflux MFS transporter EmrD
VPA0520		0.3885	Down	Multidrug effflux MFS transporter
VPA1190	*vmeZ*	2.1611	Up	Multidrug efflux RND transporter permease subunit VmeZ
VPA1191	*vmeY*	6.0915	Up	Multidrug efflux RND transporter periplasmic adaptor subunit VmeY
VPA1647		3.3176	Up	Multidrug effflux MFS transporter

### Validation of RNA-seq data by qPCR

**Figure 8:**
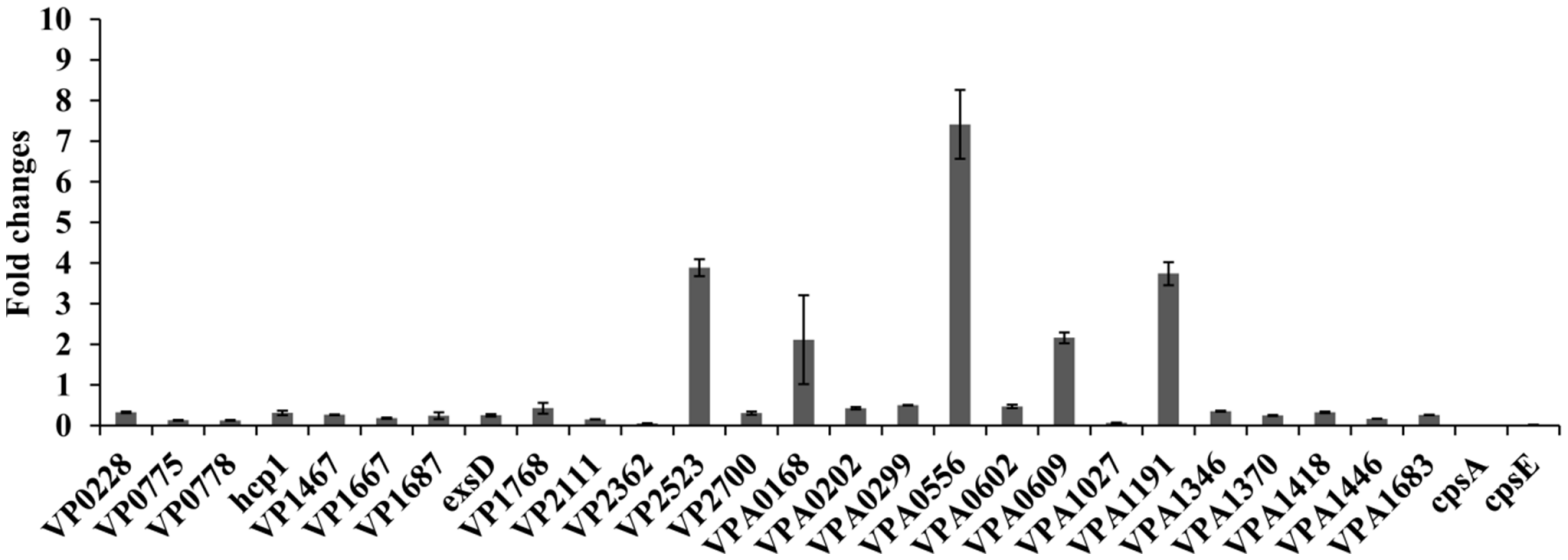
Validation of RNA-seq data by qPCR. Relative mRNA levels of target genes were compared between the bacteria grown in the presence (0.2 μg/mL) and absence of chloramphenicol. The 16S rRNA was used as the internal control.

A total of 26 DEGs were selected as the target genes (Table 2) to subject to the qPCR assays to validate the RNA-seq data. As shown in Figure 8, the results of qPCR for all the tested genes manifested consistent trends with the RNA-seq data (Table 2), confirming the reliability of RNA-seq data. In addition, the qPCR results demonstrated that transcriptional levels of *cpsA* and *cpsE* were also remarkably downregulated in response to the chloramphenicol stress.

## Discussion

Chloramphenicol has potent inhibition on bacterial protein biosynthesis via binding to the 50S subunit of ribosome (Bale et al., 2023), and thus has a significant impact on gene expression, bacterial physiology and behaviors. Addition of chloramphenicol to culture media significantly inhibited the growth and respiration of *Escherichia coli* (Smirnova et al., 2023). The biofilm formation abilities of *Klebsiella* sp., *Pseudomonas aeruginosa*, *Achromobacter* sp., *Klebsiella pneumoniae*, and *Bacillus pumilis* were significantly decreased in the presence of chloramphenicol (Liaqat et al., 2009). Chloramphenicol down-regulated flagellar gene expression and swimming and swarming motility of *Salmonella enterica* serovar Typhimurium, but up-regulated genes involved in invasion, attachment, and intracellular survival (Brunelle et al., 2014, 2017). Transcriptome analysis revealed that chloramphenicol affects global gene expression in multiple bacterial species including such as *Streptococcus pneumoniae* (Ng et al., 2003), *Staphylococcus equorum* (Heo et al., 2022), *Bacillus subtilis* (Lin et al., 2005), *Yersinia pestis* (Qiu et al., 2006), *Thermotoga maritima* (Montero et al., 2007), *Pseudomonas putida* (Fernandez et al., 2012), *Myxococcus xanthus* (Yang et al., 2019), *Enterococcus faecalis* (Tatta et al., 2023), and *Escherichia coli* (Bie et al., 2023). The genes that showed altered expression in response to chloramphenicol are involved in multiple cellular pathways including metabolism, stress response, regulation, and protein biosynthesis.

In this study, the data showed that sublethal doses of chloramphenicol remarkably inhibited the growth rate, biofilm formation, motility, c-di-GMP biosynthesis, cytoxicity and adherence activity of *V. parahaemolyticus* (Figures 1–6). The RNA-seq data disclosed that sublethal dose of chloramphenicol strikingly affected the expression of 650 genes in *V. parahaemolyticus*, of these 296 were upregulated and 354 were downregulated (Figure 7; Table 2). Majority of virulence genes (T3SS1, T6SS2, etc.), polar flagellar genes and biofilm-associated genes (*msh*, *scv., cps*, etc.) were down-regulated by sublethal dose of chloramphenicol (Figure 7; Table 2). Therefore, the reduction in functional phenotypes of *V. parahaemolyticus* is likely due to growth defect and downregulation of related gene expression. In addition, the results in this study also suggested that sublethal dose of chloramphenicol could be used as an anti-biofilm or anti-virulence regent to combat with *V. parahaemolyticus* contamination. There are currently many studies exploring the anti-virulence and anti-biofilm strategies toward bacteria (Imchen et al., 2022; Law and Tan, 2022). Findings show that some substances including cationic surfactants such as benzalkonium chloride (Park and Yoon, 2023), chemicals such as Rhein (Folliero et al., 2022) and 2,6-Di-tert-butyl-4-methylphenol (Santhakumari et al., 2018), peptides such as MSI-I (Ye et al., 2021) and PV-Q5 (Dai et al., 2023), and traditional chinese medicine such as gingerol (Shukla et al., 2021) and *Ulva fasciata* (Qiao et al., 2021) possess anti-virulence and/or anti-biofilm activity. Compared to antibiotics such as chloramphenicol, these substances have obvious advantages, such as less susceptibility to bacterial resistance. Further research is needed to discover compounds with strong applicability that can reduce the virulence and biofilm formation capacity of *V. parahaemolyticus*.

Drug efflux from bacterial cells is one of the key mechanisms of drug resistance (Stephen et al., 2022). There are five major groups of drug efflux pumps, that is, the RND family, the MF (major facilitator) family, the MATE (multidrug and toxic compound extrusion) family, the SMR (small multidrug resistance) family and the ABC (ATP binding cassette) superfamily (Stephen et al., 2022). *V. parahaemolyticus* possesses 12 RND-type efflux pumps (Matsuo et al., 2013), but only VmeYZ was induced by the sublethal dose of chloramphenicol (Table 2), suggesting that VmeYZ may play the key role in the resistance of *V. parahaemolyticus* to chloramphenicol. Moreover, VP3019, *emrD*, VPA0520 and VPA1647 encoding the MF-type pumps (Makino et al., 2003) were also upregulated by the sublethal dose of chloramphenicol (Table 2), but their roles in *V. parahaemolyticus* remain ill-defined.

Reducing antibiotic influx through OMP channels is another important mechanism of drug resistance in bacteria (Rodrigues et al., 2022). For example, in *E. coli*, OmpC is associated with β-lactams resistance, OmpF with β-lactams and fluoroquinolone resistance, and TolC, YddB, OmpX and TosA with the resistances of enrofloxacin, novobiocin, fluoroquinolones, β-lactams and globomycin (Rodrigues et al., 2022). Seventeen OMPs including OmpU, OmpN, OmpA, OmpV and OmpK are associated with the resistance of *V. alginolyticus* to erythromycin, tetracycline, kanamycin, streptomycin, nalidixic acid and chloromycetin (Xiong et al., 2010). OmpN is involved in the indole-dependent tetracycline resistance of *V. splendidus* (Zhang et al., 2020). Twelve OMP genes were regulated by the sublethal dose of chloramphenicol including the known porins such as OmpU, OmpK and OmpN (Table 2), indicating that OMPs were remodeled in the response to the chloramphenicol. However, whether these putative OMPs are associated with chloramphenicol resistance of *V. parahaemolyticus* remains unknown.

*Vibrio parahaemolyticus* has a strong ability to form biofilms, which are also closely linked to the antibiotic resistance (Mishra et al., 2023). *V. parahaemolyticus* undergoes the phase variation between the wrinkly and smooth colony phenotypes, which is based on whether the EPS production or not (Wu et al., 2022). EPS production is associated with the *cpsA-K* and *scvA-O* gene clusters that positively correlate with the biofilm formation by *V. parahaemolyticus*, but the wrinkly colony phenotype is only attributed to the expression of *cpsA-K* (Liu et al., 2022). Sublethal doses of chloramphenicol inhibited *V. parahaemolyticus* to form the wrinkly colony and biofilms (Figure 2), but no transcripts of *cps* genes were detected by RNA-seq. By contrast, six *scv* genes were remarkably low expressed in the response to chloramphenicol (Table 2). However, the results of qPCR showed that the transcriptional levels of *cpsA* and *cpsE* were notably decreased in the response to chloramphenicol (Figure 8), suggesting that the undetectable of *cps* genes probably be caused by the preparation of RNA-seq samples.

One CPS gene, VP0228, was remarkably decreased in the presence of sublethal dose of chloramphenicol (Table 2). There are 25 genes in the CPS locus (VP0214-0238) (Okura et al., 2008), and only one of them was regulated, which was likely to have no impact on the synthesis of CPS. Indeed, CPS production was not affected by sublethal dose of chloramphenicol (Figure 3). *V. parahaemolyticus* expresses dual flagellar systems: a single polar flagellum for swimming in liquid and peritrichous lateral flagella for swarming over surface (McCarter, 2004). Strains with defective polar flagellum failed to form mature biofilms, which can be restored by exogenous addition of recombinant polar flagellins (Enos-Berlage et al., 2005; Jung et al., 2019). Although lacks detailed mechanisms, lateral flagella are thought to be required for the mature biofilm formation (Yildiz and Visick, 2009). A total of 43 polar flagellar genes were remarkably downregulated in the presence of sublethal dose of chloramphenicol (Table 2). However, only 2 lateral flagellar genes were significantly differentially expressed, of these 1 was upregulated and 1 was downregulated (Table 2). Therefore, the inhibitory effect of sublethal dose of chloramphenicol on swimming probably be mainly attributed to the downregulation of polar flagellar genes, whereas on swarming may be more related to the growth inhibition of *V. parahaemolyticus*. In addition, *V. parahaemolyticus* produces two kinds of type IV pili: MSHA and ChiRP (Makino et al., 2003). MSHA and ChiRP are required for the mature biofilm formation of *V. parahaemolyticus* (Shime-Hattori et al., 2006). The RNA-seq data showed that five MSHA genes were downregulated but one ChiRP gene was upregulated in the response to sublethal dose of chloramphenicol (Table 2), indicating that chloramphenicol-dependent biofilm inhibition was closely related to the different expression of type IV pili.

c-di-GMP, a ubiquitous second messenger, is involved in controlling multiple physiological roles of bacteria including motility, adherence, biofilm formation, virulence and cell cycle progression (Jenal et al., 2017). c-di-GMP is synthesized by guanylate cyclase (DGC) carrying a GGDEF domain, whereas is degraded by phosphodiesterase (PDF) carrying either a EAL or HD-GYP domain (Jenal et al., 2017). More than 50 proteins may be involved in the metabolism of c-di-GMP in *V. parahaemolyticus* (Seshasayee et al., 2010). However, only a small portion of them were demonstrated to be required for the c-di-GMP metabolism, including the GGDEF-EAL-containing proteins, ScrG and ScrC, the EAL-containing protein LafV, and the GGDEF-containing proteins, ScrO, GefA, ScrJ and ScrL (Kim and McCarter, 2007; Ferreira et al., 2008; Kimbrough et al., 2020; Kimbrough and McCarter, 2021; Zhong et al., 2022). In this study, a total of 5 genes probably encoding DGCs or PDFs were significantly differentially expressed in the stimulation of sublethal dose of chloramphenicol (Table 2), of these 2 (VP1768 and VPA0202) were downregulated and 3 (VPA0360, VPA0556 and VPA0609) were upregulated. The clarifying functions of these genes will be beneficial for us to understand the regulatory mechanisms of sublethal dose of chloramphenicol on c-di-GMP metabolism in *V. parahaemolyticus*.

The expression levels of 28 T3SS1 genes including *exsD* were remarkably decreased in the presence of sublethal dose of chloramphenicol (Table 2). Transcription of T3SS1 genes was positively regulates by ExsA (Zhou et al., 2008). ExsD binds ExsA to block the regulatory activity of ExsA and thereby inhibiting the expression of T3SS1 genes (Zhou et al., 2010). In addition, five T3SS2 genes, one T6SS1 gene and 13 T6SS2 genes were remarkably downregulated in the response to sublethal dose of chloramphenicol (Table 2). T3SS1 is mainly involved in cytotoxicity, whereas T3SS2 predominantly contributes to enterotoxicity, and both are required for the full virulence of *V. parahaemolyticus* (Park et al., 2004; Hiyoshi et al., 2010). By contrast, T6SS1 is required for the antibacterial activity of *V. parahaemolyticus*, whereas T6SS2 mainly acts as an adherence factor (Yu et al., 2012; Salomon et al., 2013). In brief, downregulating the major virulence genes may be beneficial for *V. parahaemolyticus* to combat the killing effect of chloramphenicol.

RNA-seq data also revealed that at least 23 genes encoding putative regulators were significantly differentially expressed in the response to sublethal dose of chloramphenicol, of these 11 were downregulated and 12 were upregulated (Table 2). Some genes encode global regulators, including LysR family transcriptional regulators (VP0624, VPA0299, VPA0602, and VPA0717), TetR/AcrR family transcriptional regulators (VP1229 and VPA0053), MarR family transcriptional regulator (VP2450) and GntR family transcriptional regulator (VP1649). In addition, the sublethal dose of chloramphenicol remarkably induced the expression of CpsQ, a c-di-GMP binding regulatory protein that promotes the expression of *cpsA-K* and biofilm formation by *V. parahaemolyticus* (Ferreira et al., 2012), suggesting that this regulator may also be required for resistance to chloramphenicol stress. However, the functions of the other regulatory genes are currently unknown, and future experiments should be designed to elucidate the roles of these regulators.

In conclusion, the present data demonstrated that the growth rate, biofilm formation capacity, c-di-GMP synthesis, motility, cytoxicity and adherence activity of *V. parahaemolyticus* were remarkably downregulated by the sublethal of chloramphenicol. A total of 650 genes were significantly differentially expressed in the response to chloramphenicol, including antibiotic resistance genes, major virulence genes, biofilm-associated genes and putative regulatory genes. Majority of genes involved in polar flagellum, EPS, MSHA, T3SS1, T3SS2 and T6SS2 were downregulated. In addition, five putative c-di-GMP metabolism genes were significantly differentially expressed, which may be the reason for the decrease in intracellular c-di-GMP levels in the response to chloramphenicol. Moreover, 23 putative regulatory genes were also significantly differentially expressed due to the stimulation of sublethal of chloramphenicol, suggesting that these regulators may be involved in the resistance of *V. parahaemolyticus* to chloramphenicol stress. This work helps us to understand how chloramphenicol effect on the physiology of *V. parahaemolyticus*. However, transcriptome analysis is only a preliminary study on the adaptation mechanism of *V. parahaemolyticus* to the sublethal dose of chloramphenicol, and more research should be performed to discover the underlying molecular mechanisms especially the regulation mechanisms involved in the resistance to chloramphenicol.

## Data availability statement

The raw data of RNA-seq are deposited in the NCBI repository (accession number PRJNA8742250).

## Author contributions

MZ: Formal analysis, Investigation, Writing – original draft. LC: Formal analysis, Investigation, Writing – original draft. XiL: Investigation. XueL: Funding acquisition, Investigation. TZ: Investigation. FW: Investigation. YZ: Data curation, Formal analysis, Methodology, Supervision, Validation, Visualization, Writing – review & editing. RL: Methodology, Resources, Supervision, Validation, Writing – review & editing.

## Funding

The author(s) declare financial support was received for the research, authorship, and/or publication of this article. This study was supported by the Research Projects of Nantong Health Commission (Grant No. QN2022044).

## Conflict of interest

The authors declare that the research was conducted in the absence of any commercial or financial relationships that could be construed as a potential conflict of interest.

## Publisher’s note

All claims expressed in this article are solely those of the authors and do not necessarily represent those of their affiliated organizations, or those of the publisher, the editors and the reviewers. Any product that may be evaluated in this article, or claim that may be made by its manufacturer, is not guaranteed or endorsed by the publisher.
